# Downregulation of Tumour Necrosis Factor α Gene Expression in Peripheral Blood Mononuclear Cells Cultured in the Presence of Tofacitinib Prior to Therapy Is Associated with Clinical Remission in Patients with Rheumatoid Arthritis

**DOI:** 10.3390/cimb44050132

**Published:** 2022-04-29

**Authors:** Elena V. Tchetina, Galina A. Markova, Azamat M. Satybaldyev, Aleksandr M. Lila

**Affiliations:** 1 Immunology & Molecular Biology Laboratory, Nasonova Research Institute of Rheumatology, 115522 Moscow, Russia; g.markova2010@yandex.ru; 2 Early Rheumatoid Arthritis Department, Nasonova Research Institute of Rheumatology, 115522 Moscow, Russia; azamatsat@yandex.ru (A.M.S.); amlila@mail.ru (A.M.L.)

**Keywords:** rheumatoid arthritis, tofacitinib, gene expression, cultured peripheral blood mononuclear cells, prognostic biomarkers, TNFα

## Abstract

Rheumatoid arthritis (RA) is a chronic inflammatory disease characterized by pain, synovial hyperplasia, mononuclear cell infiltration, bone erosion and joint destruction. Efficacy of personalized therapy in RA is associated with correct choice of therapeutic agent and a possibility to predict its effect prior to treatment. Our objective was to examine the association of baseline expression of metalloproteinase (MMP)-9 and cathepsin K, which are involved in cartilage and bone degradation, as well as proinflammatory cytokines tumour necrosis factor (TNF)α and interleukin (IL)-1β in the peripheral blood mononuclear cells (PBMCs) obtained from patients with RA cultured with tofacitinib (TFCN) and remission achievement. We examined 12 tofacitinib-naïve patients with RA, with a median age of 51 years and disease duration of 37.6 months. After three months of TFCN therapy, six of these patients reached clinical remission criteria while others preserved high and moderate disease activity. PBMCs were tested prior to therapy followed by their isolation in Ficoll density gradient and cultured with 100 nM TFCN for 48 h. Gene expression analysis for MMP-9, cathepsin K, IL-1β, and TNFα was performed with quantitative real-time RT-PCR using total RNA isolated from and cultured with TFCN PBMCs compared with untreated cells. Expression of all the examined genes was significantly upregulated in those cultured with TFCN PBMCs from patients who maintained high and moderate disease activity after TFCN therapy while TNFα gene expression was significantly downregulated in patients who gained remission compared with untreated counterparts. Downregulation of TNFα gene expression in PBMCs from TFCN-naïve patients with RA cultured with TFCN prior to therapy compared with untreated counterparts might serve a prognostic biomarker for remission attainment in response to tofacitinib therapy.

## 1. Introduction

Rheumatoid arthritis (RA) is a systemic disease that involves the destruction of synovial joints [1,2]. The progression of RA is associated with the proliferation of synovial fibroblast-like synoviocytes resulting in pannus formation and release of various bone- and articular cartilage degrading molecules [3]. The invasion of synovial tissue by macrophages and activated lymphocytes occurs simultaneously. T-lymphocytes are capable of producing pro-inflammatory cytokines, predominantly from the tumour necrosis factor (TNF) and interleukin (IL) superfamilies, and growth factors. B-lymphocytes are associated with the production of autoantibodies, such as rheumatoid factor (RF) and anti-cyclic citrullinated peptide antibodies (ACPA) [1]. Later, the invasion of articular cartilage and bone by degrading enzymes, primarily matrix metalloproteinases (MMPs) and cathepsins, activated by proinflammatory cytokines, produces these tissues’ destruction [4]. RA therapy involves non-steroidal anti-inflammatory drugs (NSAIDs) and biological drugs to prevent tissue destruction and suppress the disease activity, inflammation, and pain. However, not all patients responded equally to therapy, while clinical remission was observed only in 30–60% of patients with RA [5]. Therefore, the prognosis of personal response to treatment prior to therapy is of particular importance.

Tofacitinib (TFCN) is a recent disease-modifying anti-rheumatic drug (DMARD) used for monotherapy in patients with RA and represents a Janus kinase (JAK)1/3 inhibitor that limits the activity of intracellular tyrosine kinases [6]. TFCN therapy outcomes are significantly higher compared with methotrexate in the treatment of naive patients with RA or subjects with an inadequate response to biological DMARDs [7]. However, TFCN might be not applicable for patients at risk of infection or thromboembolic disturbances as the drug inhibits proinflammatory cytokine-signalling pathways that are involved in host defence mechanisms [8]. Therefore, it is important to identify responders to TFCN prior to therapy.

Since peripheral blood is the most easily accessible tissue while gene expression is the earliest cellular response of the body to environmental changes [9], gene expression analysis in peripheral blood mononuclear cells (PBMCs) might have a potency in assessing a patient’s response to treatment. Indeed, recently we have demonstrated that clinical remission attainment in patients with RA treated with TFCN was associated with lower baseline expression of genes involved in energy generation (pyruvate kinase and succinate dehydrogenase) compared with other examined subjects, while non-responsiveness to TFCN was accompanied by high baseline expression of these genes [10].

Another approach for the prediction of RA patients’ response might involve the assessment of the drug effects in PBMCs cultured in vitro prior to treatment. It is well established that degradation of articular cartilage and bone in RA are associated with the excessive production of matrix metalloproteinases (MMPs) and osteolytic enzymes, such as MMP-9 and cathepsin K [11,12], which were detected in the serum and synovial fluid of these patients [13,14]. In addition, serum concentrations of cathepsin K were associated with radiological joint destruction while MMP-9 expression can be upregulated by proinflammatory cytokines TNFα and IL-1β [15,16,17] that are subjected to change in response to RA therapy [18,19,20]. Therefore, here we hypothesized that gene expression analyses performed in the peripheral blood mononuclear cells from patients with RA cultured with TFCN prior to therapy might represent a useful approach for personalized prediction of TFCN therapy efficacy.

## 2. Materials and Methods

### 2.1. Ethics Statement

Our study was performed in accordance with the Declaration of Helsinki. The study protocol (No. 13, dated 4 June 2015) was approved by the Local Human Research Ethics Committee, and informed consent was obtained from all subjects.

### 2.2. Patients

The study included 12 female patients with RA aged ≥ 20 years (mean age 51.0 ± 14.2 years), with median disease duration 37.6 months, who did not receive tofacitinib previously. Six of these patients with RA gained remission after 3 months of TFCN therapy while six patients maintained high (3 out of 6) or moderate (3 out of 6) disease activity. All patients were diagnosed based on the American College of Rheumatology (ACR) classification criteria. TFCN was prescribed to all the examined patients owing to refractoriness to methotrexate (at a dose of 20–25 mg/week), a combination therapy with methylprednisolone (8 mg per day) (in 3 of 12 patients), and hydroxychloroquine (0.2 mg/day) (in one of 12 patients). All patients received TFCN (5–10 mg twice a day) during 3 months of follow-up.

All patients were evaluated by the same rheumatologist during follow-up. The inclusion criteria for this study were: confirmed diagnosis of RA based on the ACR-EULAR 2010 criteria; high and medium disease activity (DAS28 > 3.2); patient’s age between 20 and 80 years; non-response and/or intolerance of former therapy; and adequate contraceptive procedures in patients with reproduction potential. The exclusion criteria included: pregnancy and lactation; active infections; organ or haematological disturbances; demyelinating, cancerous or precancerous conditions; alcohol or drug addiction; allergic reactions to proteins; and immunisation with vaccines four weeks before study onset. ACR criteria for clinical remission based on the simplified 28-joint score (DAS28 < 2.6) were used for remission designation.

### 2.3. Demographic, Clinical, and Immunological Evaluations

The following data were obtained at baseline and at 3 months: age, sex, disease duration, Steinbrocker’s radiographic stage and the DAS28-ESR score. Nephelometric analysis was performed using a BN-ProSpec analyser (Marburg, Germany) to measure serum C-reactive protein (CRP, cut-off value 5 mg/L) and immunoglobulin M rheumatoid factor (RF, a standard cut-off value 15 mU/L) concentrations. Anti-citrullinated protein autoantibodies (ACPA) were measured using an enzyme-linked immunosorbent assay (ELISA) kit according to the manufacturer’s recommendations (cut-off level 5 U/mL for antibody positivity) (Axis Shield Diagnostics Limited, Dundee, UK).

### 2.4. Peripheral Blood Fractionation

Peripheral blood (10 mL) was collected in vacutainer tubes containing ethylenediaminetetraacetic acid (EDTA) (Ailiton, Dubna, Russia). The blood samples were taken in a standardised manner in the morning (between 07:00 AM and 09:00 AM). Whole blood fractionation was performed using a Ficoll density gradient. Peripheral blood mononuclear cells (PBMCs) were collected and washed twice in phosphate-buffered saline (PBS).

### 2.5. Cell Viability Test

Cell viability of PBMCs from patients with RA was assessed by 0.2% Trypan blue staining after 48 h of TFCN treatment. Cell viability was considered satisfactory in the case where the same number of viable cells was observed in control cells (cultured without TFCN) or PBMCs treated with TFCN. The examined concentrations of TFCN (10 nM, 100 nM or 500 nM) were non-toxic as cell viability was not affected compared with untreated cells. As at 100 nM, tofacitinib demonstrated the most pronounced results in gene expression analyses, we used this concentration in all the experiments.

### 2.6. Peripheral Blood Mononuclear Cells Culture

PBMCs were seeded 10^6^ cells/mL in a 96-well plate and cultured in RPMI-1640 medium (Gibco, Life Technologies, Inc., Paisley, UK) containing 25 mmol/L HEPES buffer, pH 7.4, supplemented with 10% (*v*/*v*) of heat-inactivated foetal bovine serum (FBS, Gibco, Life Technologies, Inc., Paisley, UK), 100 U/mL penicillin, 100 μg/mL streptomycin and 150 μg/mL gentamicin sulphate at 37 °C in the presence of 5% CO_2_ for 48 h. After change of the growth medium, cells were subjected to fasting for 24 h in the same medium without serum. After medium change, cells were supplemented with fresh medium without serum either containing 100 nM tofacitinib citrate (Pfiser Inc., Freiburg, Germany) or the same medium without TFCN (Control). After 1 h of incubation, 10% (*v*/*v*) FBS was added to each well and cells were further incubated another 48 h. Cells were harvested and total RNA was isolated immediately. All experiments were conducted in triplicate.

### 2.7. Total Ribonucleic Acid (RNA) Isolation and Reverse Transcriptase (RT) Reaction

Total RNA was isolated from each well using Extract RNA Reagent (Evrogen, Moscow, Russia). The RT-reaction was performed using a kit that contains M-MLV Reverse Transcriptase, random hexanucleotide primers and uses total RNA, according to the manufacturer’s recommendations (Evrogen, Moscow, Russia).

### 2.8. Real-Time Quantitative Polymerase Chain Reaction (PCR)

The following pre-made primers and probes were used to perform the TaqMan assay (Applied Biosystems, Foster City, CA, USA): TNFα (Hs00174128_m1), IL-1β (Hs00174097_m1), MMP-9 (Hs00234579_m1), and cathepsin K (Hs00987255_m1). β-Actin was used as an endogenous control. Gene expression was quantified using the Quant Studio 5 real-time PCR System (Applied Biosystems, Foster City, CA, USA) as described previously [21]. Briefly, 1 μL of RT product was subjected to real-time PCR in a total reaction mixture (15 μL) containing 7.5 μL of TaqMan Universal PCR Master Mix (Applied Biosystems, Foster City, CA, USA), 900 nM sense and antisense primers, 50 nM probe, and template cDNA. After a single step at 50 °C for 2 min and initial activation at 95 °C for 10 min, the reaction mixtures were subjected to 40 amplification cycles (15 s at 95 °C for denaturation and 1 min of annealing and extension at 60 °C). Relative mRNA expression was determined using the ΔΔC_T_ method per the manufacturer’s guidelines (Applied Biosystems, Foster City, CA, USA). The ΔC_T_ value was calculated by subtracting the C_T_ value for the housekeeping gene from the C_T_ value for each sample. Subsequently, the ΔΔC_T_ value was calculated by subtracting the ΔC_T_ value of each control from the ΔC_T_ value observed in each treated by TFCN sample.

### 2.9. Statistical Analysis

Quantitative data were expressed as medians (IQR, 25th; 75th percentiles). All statistical analyses were performed using the Statistica software package (version 12, StatSoft Inc., Tulsa, OK, USA). The Mann–Whitney and Wilcoxon signed-rank tests were used for statistical processing of the results. *p* value ≤ 0.05 was considered statistically significant. All statistically significant differences are indicated with an asterisk (*) or (^#^).

## 3. Results

### 3.1. Clinical and Immunological Characteristics and Therapeutic Response in Patients with Rheumatoid Arthritis at Baseline and after Three Months of Tofacitinib Therapy

The mean duration of RA in the examined patients was 37 months, which varied from 4 to 180 months. At baseline, 11 patients presented with Steinbrocker’s radiographic stage II disease, and one patient had stage III RA. One patient was seronegative for ACPA, and five were negative for RF; the remaining patients (11 and 7 patients, respectively) were seropositive for these biomarkers. At study commencement, seven patients with RA had high disease activity (DAS28 > 5.1) and five had moderate activity (3.2 < DAS28 < 5.1). We did not observe significant differences in the majority of baseline characteristics between patients who achieved remission (subgroup 1) and other patients with RA (subgroup 2) (Table 1). However, subgroup 2 patients demonstrated higher number of tender joints (*p* = 0.04) and a trend for higher RF values (*p* = 0.07) compared with patients of subgroup 1 at baseline.

After 3 months of follow-up, patients from both subgroups showed a significant decrease in the number of swollen joints (*p* = 0.03), while the absence of tender joints was observed only in patients who gained remission (Table 1). At the same time, subgroup 2 patients demonstrated a trend for the increase in ESR (*p* = 0.06). In addition, we noted that both patient subsets responded to TFCN treatment (ΔDAS 28 > 1.2). However, ΔDAS 28 values from patients who gained remission was significantly higher compared with subgroup 2 subjects.

### 3.2. Gene Expression Analyses in the Peripheral Blood Mononuclear Cells Cultured with Tofacitinib Prior to Therapy

**Figure 1:**
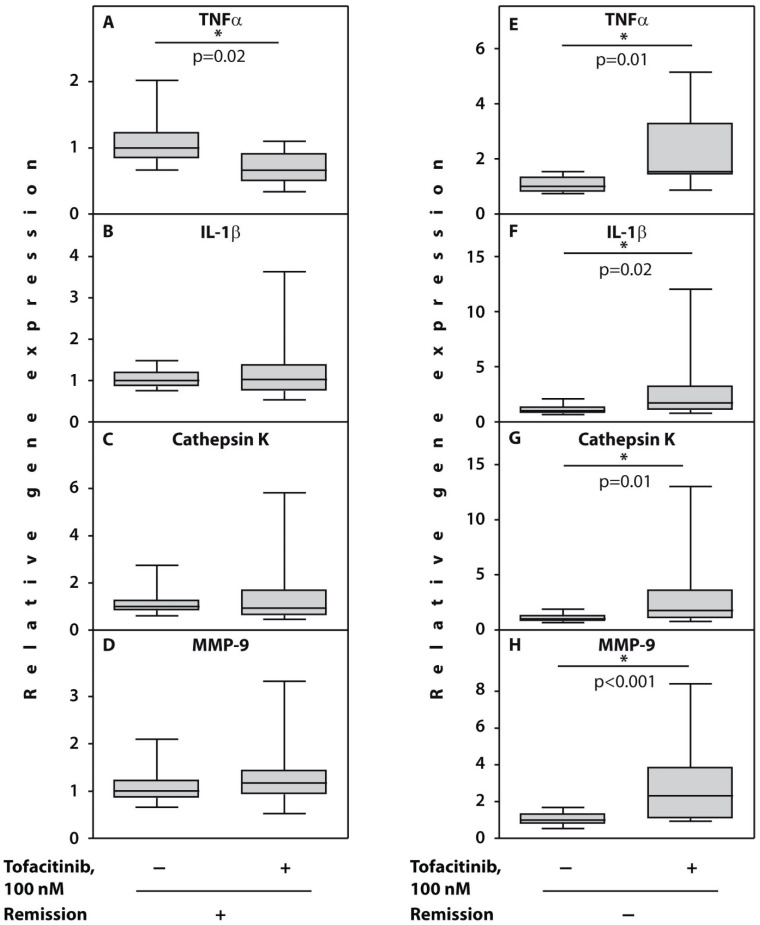
Relative gene expression in PBMCs from patients with RA prior to therapy cultured with tofacitinib during 48 h. (**A**–**D**) patients who gained remission (*n* = 6); (**E**–**H**) no remission (*n* = 6) was observed. Asterisks (*) indicate significant differences from control cells cultured without TFCN (Wilcoxon matched pairs test). Abbreviations: TNFα, tumour necrosis factor alpha; IL-1β, interleukin 1 beta; MMP-9, metalloproteinase 9.

Significant upregulation in expression of all the examined genes was observed in cultured with TFCN PBMCs compared with untreated control cells from patients (*n* = 6) who maintained high or moderate disease activity after 3 months of TFCN treatment (Figure 1E–H). In particular, MMP-9, TNFα, and IL-1β gene expressions were upregulated in five out of six patients, while cathepsin K gene expression was upregulated in all the examined patients from subgroup 2.

In contrast, in the subgroup 1 patients (*n* = 6) who gained remission after 3 months of follow-up, significant downregulation of TNFα gene expression was noted in those cultured with TFCN PBMCs from all the examined subjects compared with untreated controls (Figure 1A). Other examined genes demonstrated variable responses. For example, IL-1β, MMP-9, and cathepsin K gene expression in cells cultured with TFCN was downregulated in three out of six patients while another three demonstrated upregulation of these gene expressions (Figure 1B–D) compared with non-treated controls.

**Figure 2:**
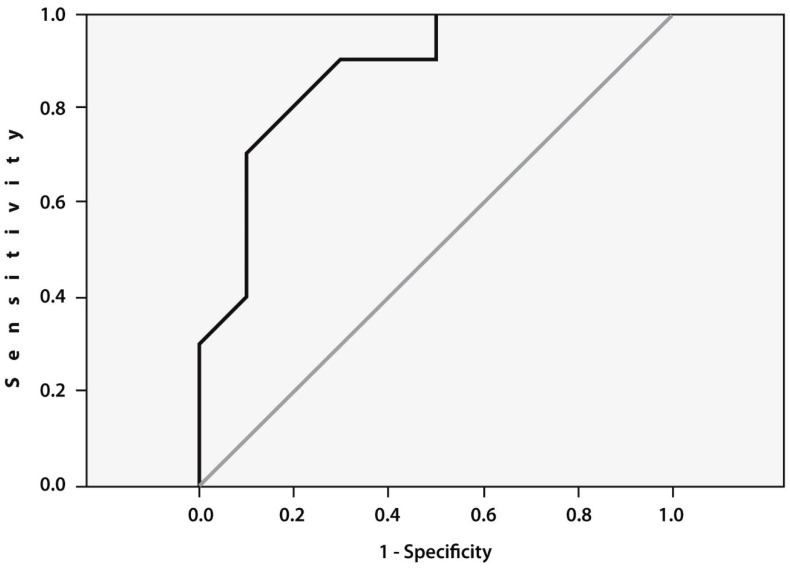
Area under the curve (AUCs) between the baseline TNFα gene expression in the peripheral blood mononuclear cells of patients with RA cultured with tofacitinib for 48 h prior to therapy. Receiver-operating characteristic (ROC) curve for the expression of TNFα (AUC = 0.875, 95% CI (0.718–1.000), *p* = 0.005).

To assess the prognostic value of TNFα gene expression, we performed ROC curve analysis (Figure 2), which confirmed significant association between expression of this gene examined prior to TFCN treatment with the likelihood of remission development after therapy. The cut-off value for TNFα gene expression was 1.17 (AUC = 0.875, 95% CI (0.718–1.000), *p* = 0.005).

## 4. Discussion

The number of therapeutic targets for the treatment of RA is constantly growing. As a result, more therapeutic agents specifically pursuing these targets become clinically available [22]. Clinical studies show that various drugs are not equally efficient in the treatment of patients with RA; therefore, responders and non-responders to therapy are required to be identified [23]. Here, we analysed changes in the expression of genes related to inflammation and joint destruction in patients who gained remission after treatment with tofacitinib for 3 months and other responders to the drug (ΔDAS28 > 1.2) who failed to achieve remission and maintained high or moderate disease activity.

We demonstrated that remission attainment in patients with RA treated with TFCN is associated with a significant decrease in TNFα gene expression in PBMCs cultured with TFCN prior to therapy. Our results are also supported by animal studies, which demonstrate that TFCN suppressed TNFα production in cultured blood cells [24]. On the other hand, our results explain the absence of the difference in TNFα expression between responders and non-responders previously observed in studies with T cells from patients with RA cultured with TFCN in the same concentration [25] as we observed that TNFα gene expression was downregulated only in patients who gained remission and not in other responders. Mechanistically, in cellular assays, TFCN preferentially inhibits cytokine signalling that requires the JAK1/3-STAT pathway [26]. Although TNFα does not directly signals by JAK-STAT, tofacitinib has been previously shown to be capable of downregulating cell responses to TNFα partly by suppressing TNF-IFNβ-JAK-STAT1 autocrine loop [27]. Therefore, as TNFα operates upstream IFNβ-JAK-STAT signalling, and downregulation of this pathway shortly (in 48 h) after drug application in the most sensitive responders that are capable of developing remission is reasonable.

In contrast, PBMCs from patients that failed to develop remission after 3 months of TFCN therapy demonstrated a significant increase in baseline TNFα, IL-1β, MMP-9, and cathepsin K gene expressions when cultured with this drug. Upregulation of MMP-9 expression in response to inhibition of JAK was also previously observed in macrophage cell cultures [28]. These results are supported by changes in clinical indices in response to treatment as upregulation of TNFα, MMP-9, cathepsin K, and IL-1β gene expressions in a subgroup 2 patients with less pronounced response to treatment was associated with a trend of higher values of RF at baseline and ESR after 3 months, as well as maintenance of several swollen and tender joints at the end of the follow-up compared with subgroup 1 patients. Our results are in line with previous observations of the importance of the examined gene expressions for inflammation and bone resorption activities in RA [29,30,31].

Interestingly, although it is generally accepted that ESR and CRP are performed similarly in observational studies [32], in the case of TFCN, we observed a trend for reciprocal changes involving downregulation of CRP and upregulation of ESR in subgroup 2 RA patients with a less prominent response to the drug.

## 5. Conclusions

A significant downregulation of TNFα gene expression in PBMCs cultured with tofacitinib for 48 h compared with untreated counterparts prior to therapy is associated with patients’ remission attainment after treatment. In contrast, a significant upregulation of TNFα, IL-1β, MMP-9, and cathepsin K gene expressions might point to less prominent responses to the drug. Our original approach of therapy efficacy estimation in patients with rheumatoid arthritis by assessing changes in gene expression in the peripheral blood mononuclear cells cultured in vitro with the drug before treatment might be important for personalized medication selection before its administration in a clinical setting.

## Figures and Tables

**Table 1:** Characteristics of patients who achieved remission (*n* = 6) and other examined patients with rheumatoid arthritis (*n* = 6) prior to and after tofacitinib therapy.

	Subgroup 1 Patients Who Achieved Remission (*n* = 6) DAS28 < 2.6		*p*	Subgroup 2 Other Examined Patients with RA (*n* = 6)		*p*	*p*′ Prior to Therapy
	Baseline (*n* = 6) Me [IQR]	After 3 Months (*n* = 6) Me [IQR]		Baseline (*n* = 6) Me [IQR]	After 3 Months (*n* = 6) Me [IQR]		
Age, years ^#^	46.5 [29.5; 59.5]			60 [41.5; 69]			0.18
Disease duration, months ^#^	30 [16.5; 108]			21 [6; 39]			0.42
IgM RF, mU/L ^#^	13 [8; 18]			48 [12; 75]			0.07
ACPA, U/mL ^#^	30 [14; 74.5]			35.5 [25.5; 40]			0.69
CRP, mg/mL	18.6 [2.4; 98]	3.9 [0.25; 8.6]	0.06	17.5 [8.7; 83.2]	5.2 [1; 28.5]	0.31	0.93
ESR, mm	30 [14; 74.5]	13 [8; 18]	0.12	35.5 [25.5; 40]	48.0 [12; 75]	0.43	0.69
DAS 28	4.85 [3.6; 6.2]	1.95 [1.3; 2.2]	0.03 *	6.2 [5.3; 7.0]	4.8 [3.9; 5.4]	0.03 *	0.09
ΔDAS 28 ^#^		2.77 [1.9;4.2]			1.4 [0.9; 2.1]	0.01 *	
Number of swollen joints	5.5 [3; 11.5]	0 [0; 0.5]	0.03 *	12.5 [7; 17]	2 [1; 8.5]	0.03 *	0.06
Number of tender joints	4 [2.5; 10.5]	0 [0; 0]	-	13.5 [7; 20.5]	6.5 [1.5; 15.5]	0.06	0.04 ^#^

Asterisks (*) indicate significant differences between the examined subgroups of patients. (Wilcoxon signed-rank test); (^#^), Mann–Whitney U test; IQR, interquartile range.
